# Rapid onset of effect of benralizumab in a severe eosinophilic and allergic asthma patient with allergic bronchopulmonary aspergillosis

**DOI:** 10.1002/rcr2.1167

**Published:** 2023-05-25

**Authors:** Arvindran Alaga, Khairil Ashraff, Nurul Hana Din Khan

**Affiliations:** ^1^ Respiratory Department Hospital Sultanah Bahiyah Alor Setar Malaysia

**Keywords:** allergic asthma, allergic bronchopulmonary aspergillosis, benralizumab, case report, eosinophilic asthma

## Abstract

There is limited data on the use of benralizumab in patients with severe asthma, who have allergic bronchopulmonary aspergillosis (ABPA). We report the case of a 65‐year‐old woman with combined severe eosinophilic and allergic asthma, who presented with refractory respiratory symptoms, hypereosinophilia and high immunoglobulin E (IgE) level. The patient had consistently poor Asthma Control Test (ACT) scores, despite a maximum dose of inhalation therapy. Upon further investigations, she was diagnosed with concomitant ABPA. The patient was started on oral prednisolone and itraconazole, but her symptoms persisted. She was then started on subcutaneous omalizumab, but switched to benralizumab after developing a severe allergic reaction. The patient experienced rapid clinical improvements after the first dose of subcutaneous benralizumab. Benralizumab demonstrated a significant role in reducing the exacerbation rate and oral corticosteroid use in this patient, as well as improving lung function, asthma control, and quality of life measures.

## Graphical abstract

Case report of a 65‐year‐old woman with combined severe eosinophilic and allergic asthma, who was later diagnosed with concomitant allergic bronchopulmonary aspergillosis. Benralizumab demonstrated a significant role in reducing the exacerbation rate and oral corticosteroid use in this patient, as well as improving lung function, asthma control, and quality of life measures.

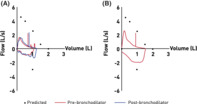

## INTRODUCTION

Therapeutic effects of benralizumab, an anti‐interleukin‐5 receptor subunit α (IL5Ra) monoclonal antibody, has been demonstrated in severe eosinophilic asthma patients, for both allergic and non‐allergic phenotypes.^1^ Patients with positive skin prick test (SPT) have been shown to achieve significant improvements in exacerbation rate, ACT score and daily short‐acting β_2_‐adrenergic agonists (SABA) use with benralizumab, compared to patients with negative SPT.^1^ Complete prevention of asthma exacerbations may be achievable from a 6‐month benralizumab regimen.^1^ However, there is limited data available from clinical trials on the use of benralizumab in treating severe asthma patients with allergic bronchopulmonary aspergillosis (ABPA). We report a case of a patient with combined severe eosinophilic and allergic asthma and newly‐diagnosed allergic bronchopulmonary aspergillosis (ABPA), who showed rapid improvements after the first dose of benralizumab.

## CASE REPORT

The patient was a 65‐year‐old female with severe eosinophilic and allergic asthma. The patient had been diagnosed with asthma since 2012 at a local health facility before being referred to a tertiary centre in 2016 as her asthma became poorly controlled. Between 2016 and 2018, the patient experienced exacerbations up to three times per week, with monthly casualty visit for nebulisation and requiring hospital admission up to 1–2 times per month. The patient's Asthma Control Test (ACT) scores during this period were as low as 13–16 (out of a maximum possible score of 25).

In 2021, she presented with refractory respiratory symptoms (specifically dyspnoea, wheezing, and intermittent cough), hypereosinophilia and high immunoglobulin E (IgE) level. The patient showed consistently poor ACT scores, despite being on multiple inhalers from 2016 to 2021, including inhaled corticosteroids (ICS), long‐acting beta‐agonists (LABA) + ICS, and long‐acting muscarinic antagonists (LAMA). The patient had also received oral corticosteroid (OCS) at a dose of 5–10 mg/day for 4 years.

**FIGURE 1:**
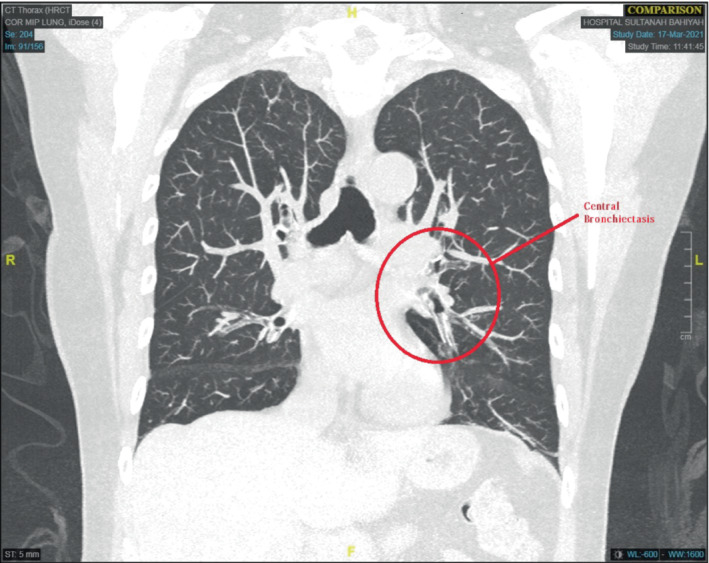
Computed tomography (CT) scan of the thorax, depicting central bronchiectasis due to allergic bronchopulmonary aspergillosis (ABPA).

A computed tomography (CT) scan of the thorax showed central bronchiectasis, which is associated with ABPA (Figure 1). Her total serum IgE level was elevated to 5000 kU/L, and IgE specific for *Aspergillus fumigatus* was elevated moderately to 2.38 kUA/L. Based on the International Society for Human and Animal Mycology (ISHAM) criteria, the patient was diagnosed with concomitant ABPA.^2^

The patient started treatment with oral prednisolone (0.5 mg/kg daily for 14 days, followed by 0.5 mg/kg every other day, then further tapered and finally discontinued at 4 months) and itraconazole (200 mg BD for 16 weeks), but her symptoms remained persistent and her IgE levels remained elevated after 4 months of treatment. The patient was then started on subcutaneous omalizumab (300 mg), but developed a severe allergic reaction, including skin rashes and worsening dyspnoea, after the first dose.

**FIGURE 2:**
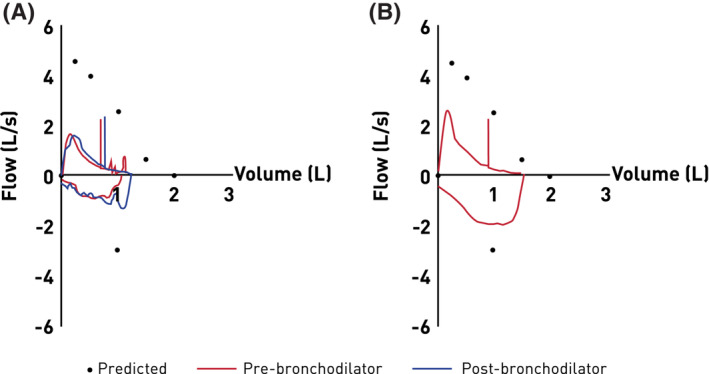
Spirometry with bronchodilator (A) pre‐ and (B) post‐benralizumab.

A decision was taken to switch the patient to subcutaneous benralizumab (30 mg) every month for first 3 months, and then every 2 months. The patient experienced rapid clinical improvements after the first dose of benralizumab; her respiratory symptoms were significantly improved, and peripheral eosinophilia was rapidly reduced from 540 to 0 cells/μL within a week post‐injection and remained stable. Her ACT score improved from a range of 10–12 before benralizumab, to a score of 22 and above. Spirometry test also showed improved lung condition, with most spirometry parameters before benralizumab improving after initiating benralizumab (Figure 2; Table 1). The patient's IgE level reduced to 3500 kU/L after 2 months of benralizumab.

**TABLE 1:** Spirometry parameters pre‐ and post‐benralizumab.

Parameters	Pre‐benralizumab				Post‐benralizumab	
	Pre‐bronchodilator		Post‐bronchodilator		Pre‐bronchodilator	
	Actual	% Predicted	Actual	% Predicted	Actual	% Predicted
FVC (L)	1.13	56	1.24	62	1.52	77
FEV1 (L)	0.71	43	0.78	47	0.90	55
FEV1/FVC (%)	63	–	63	–	59	–
FEF 25% (L/s)	1.16	28	1.50	37	1.26	31
FEF 75% (L/s)	0.21	30	0.24	35	0.20	30
FEF 25–75% (L/s)	0.39	21	0.51	27	0.42	23
FEF Max (L/s)	1.64	35	1.60	34	2.68	58
FIVC (L)	1.07	–	1.34	–	1.58	–
FIF Max (L/s)	0.98	–	1.34	–	1.97	–

Abbreviations: FEF Max: maximum instantaneous flow; FEF, forced expiratory flow; FEV1, forced expiratory volume in 1 s; FIF Max, maximum forced inspiratory flow; FIVC, forced inspiratory vital capacity; FVC, forced vital capacity.

The patient was able to reduce her asthma reliever use from 4 to 6 times a week in the 6‐month period preceding the first benralizumab dose, to only once a month. She also successfully tapered off her OCS use. For this patient, benralizumab allowed reduction of OCS, while improving control of asthmatic exacerbation.

## DISCUSSION

To our knowledge, this is the first case report of ABPA achieving positive treatment outcomes with benralizumab in Southeast Asia. In this report, dramatic improvements were observed in the patient's symptoms, lung function, peripheral eosinophil count, and IgE level.

ABPA is a hypersensitivity response to fungal (*A. fumigatus*) infection of the lungs, and is commonly seen in asthmatic and immunocompromised patients.^3^ The prevalence of ABPA in asthmatics have been reported at 12.9%, and the disease tends to worsen patients' respiratory symptoms.^2^, ^4^ The diagnostic criteria of ABPA include bronchial asthma, immediate cutaneous hypersensitivity to *A. fumigatus*, elevated total and *A. fumigatus*‐specific serum IgE, pulmonary opacities, bronchiectasis, peripheral blood eosinophilia and presence of serum precipitins or raised IgG to *A. fumigatus*. However, these criteria are non‐specific to ABPA, and patients at various stages of ABPA may not fulfil all criteria.^4^

ABPA is primarily managed with glucocorticoids as an anti‐inflammatory agent. Although most patients would respond to standard doses of glucocorticoids, some patients, such as our patient, may not adequately respond to oral glucocorticoid therapy.^5^ In recent years, systematic reviews and case reports have reported the potential benefit of biologics, including benralizumab, for the treatment of ABPA in patients with asthma or cystic fibrosis.^6^, ^7^, ^8^, ^9^, ^10^, ^11^, ^12^ However, the efficacy and safety of benralizumab in ABPA remains to be fully studied.

In this case study, benralizumab demonstrated a significant role in reducing the exacerbation rate and OCS use, as well as improving lung function, asthma control, and quality of life measures. We postulate that the treatment acted upon eosinophilic asthma as well as ABPA. This is because, despite full course of ABPA treatment, the patient still experienced frequent exacerbation and persistently high IgE levels (>5000 kU/L, when we anticipate ~35% reduction after treatment). This case report suggests benralizumab's potential as an alternative treatment in ABPA management. Prospective randomized trials are warranted to assess benralizumab's efficacy and safety in treating severe asthma with concomitant ABPA.

## AUTHOR CONTRIBUTIONS

All authors contributed, reviewed and approved the final manuscript.

## CONFLICT OF INTEREST STATEMENT

Arvindran Alaga, Khairil Ashraff, and Nurul Hana Din Khan declare no conflict of interest.

## FUNDING STATEMENT

The development of this manuscript was funded by AstraZeneca Malaysia.

## ETHICS STATEMENT

The authors declare that appropriate written informed consent was obtained for the publication of this manuscript and accompanying images.

## Data Availability

The data that support the findings of this study are available on request from the corresponding author. The data are not publicly available due to privacy or ethical restrictions.
